# Genetics and Genomics of Reproductive Medicine

**DOI:** 10.3390/genes12101612

**Published:** 2021-10-14

**Authors:** Rossella Tomaiuolo

**Affiliations:** Vita-Salute San Raffaele University, 20132 Milan, Italy; tomaiuolo.rossella@hsr.it

The rapidity of innovations has meant that reproductive medicine today represents clear example of how complex but essential an adaptation of clinical practice and laboratory techniques to new knowledge is in the context of the dynamic evolution of medicine. For this purpose, the Special Issue “Genetics and genomics in Reproductive Medicine” is designed to discuss the impact of new advances in reproductive medicine and to support the improvement of diagnostic and operational processes in genetics laboratories.

Infertility is a complex disorder of the reproductive system, characterized by the inability to get pregnant after more than 12 months of regular and unprotected sexual intercourse, affecting more than 75 million couples of reproductive age in the world. All the articles cover fundamental aspects of healthy reproduction, from female and/or male fertility to antenatal diagnosis: it consists of nine original research articles, two reviews and one perspective covering various aspects of genetics and genomics of reproductive medicine.

Genetics now play an essential role in all stages of the reproductive path, from the diagnostic approach to the choice of more complex therapies: numerous research advances in genetics have increased reproductive medicine’s success. For example, the rapid progress of the technologies applied to molecular diagnosis provides several diagnostic and screening options for the identification of genetic and chromosomal alterations related to infertility [1], non-syndromic infertility [2] or recurrent pregnancy loss [3]. However, while female infertility has been extensively investigated, only recently, attention is being paid to the etiological and pathophysiological causes related to male infertility. At this scope, Pagliuca et al. [4] investigate the implication of infection on the main sperm parameters.

Alongside advanced biotechnology applications, identifying novel molecular biomarkers is a promising approach to improve the success of assisted procreation techniques (PMAs) or reduce the risk of repeated abortion. Here, Rocca MS et al. [5] examine the role of the telomeres as a predictive factor for embryo quality in the setting of medically assisted reproduction. In particular, the sperm telomere length (STL) is a novel biomarker of male fertility, as telomeres have recently been shown also to include a long non-coding RNA (lncRNA) known as TERRA (telomeric repeat-containing RNAs).

Further, Shilenkova Y.V. et al. [6] propose to correlate the impact of age and serum anti-Müllerian hormone (AMH)/follicle-stimulating hormone (FSH) levels on the number of cumulus-oocyte complexes (COCs) retrieved with controlled ovarian hyperstimulation (COH) from female carriers of reciprocal and Robertsonian translocation. In this volume, additional potential biomarkers have been described concerning pregnancy outcome and recurrent pregnancy loss: in a multi-center prospective cohort study on mosaic embryos, Zhang Y et al. reported the pregnancy outcome providing information from prenatal genetic testing [7]; An H et al. investigate whether glycoprotein 6 (GP6) gene polymorphisms are a risk factor for recurrent pregnancy loss [8].

The positive effects of these continuous improvements can also be found in prenatal diagnosis [9,10,11] to identify genetic diseases and chromosomal alterations during the antenatal stage; available options range from non-invasive prenatal screening (NIPT) to targeted assessment of at-risk couples.

Attention to fertility must be placed on infertile couples and in the current socio-economic context. It seems to us that this may take on particular importance at a time when Medicine, of appropriateness and precision, cannot be separated from socio-health and economic evaluations. The multidisciplinary approach, continuous research and innovation of laboratory methods allow patients and couples with fertility problems, spontaneous abortions and endocrinological disorders to affect reproductive function and high-quality diagnostic-therapeutic paths. Consequently, if on the one hand, it is necessary to continue to invest in clinical and laboratory research to improve the reproductive "outcome", on the other hand, it is essential to proceed quickly with the integration of the legislative apparatus, as well evidenced by the contribution of D’Argenio et al. [12].

In conclusion, the current Special Issue intends to have a broad look at the various segments that contribute to healthy reproduction, having the field of genetics and genomics as a preferential angle of observation. These advances set the stage for further investigations that will improve the outcome of healthy reproduction.
